# A Transcranial Stimulation Intervention to Support Flow State Induction

**DOI:** 10.3389/fnhum.2019.00274

**Published:** 2019-08-08

**Authors:** Joshua Gold, Joseph Ciorciari

**Affiliations:** ^1^Centre for Mental Health, Swinburne Neuroimaging (SNI), Swinburne University of Technology, Hawthorn, VIC, Australia; ^2^Department of Psychological Sciences, Swinburne University of Technology, Hawthorn, VIC, Australia

**Keywords:** flow, psychophysiology, tDCS, neuromodulation, decision making

## Abstract

**Background:** Flow states are considered a positive, subjective experience during an optimal balance between skills and task demands. Previously, experimentally induced flow experiences have relied solely on adaptive tasks.

**Objective:** To investigate whether cathodal transcranial direct current stimulation (tDCS) over the left dorsolateral prefrontal cortex (DLPFC) area and anodal tDCS over the right parietal cortex area during video game play will promote an increased experience of flow states.

**Methods:** Two studies had participants play Tetris or first-person shooter (FPS) video games while receiving either real tDCS or sham stimulation. Tetris recruited 21 untrained players who infrequently played video games while the 11 FPS participants played FPS frequently. Flow experience was assessed before and after stimulation.

**Results:** Compared to sham stimulation, real stimulation increased flow experience for both untrained Tetris and trained FPS players. Improved performance effects were only seen with untrained groups.

**Conclusion:** Cathodal and anodal tDCS over the left DLPFC and right parietal areas, respectively may encourage flow experiences in complex real-life motor tasks that occur during sports, games, and everyday life.

## Introduction

Flow, or optimal experience is a “holistic response” which results from a harmony found between all the states of consciousness and the individuals' skills matching their goals (Csikszentmihalyi, 1990). According to Csikszentmihalyi's (1990, 1997) flow theory, the flow state relates to the skill set perceived to be possessed by the individual relative to the perceived challenges of the activity. Challenges can be considered as “opportunities for action” thus flow is produced by any situation that requires skill (Csikszentmihalyi and Nakamura, 1999; Nakamura and Csikszentmihalyi, 2014). One of the leading neurocognitive theoretical models of flow purported by Dietrich (2004) denotes a state of transient hypofrontality, which enlists the full support of the implicit system to execute a task at optimal output (maximum skill/maximum efficiency) while the majority of the online executive function of the prefrontal cortices are inhibited (Dietrich, 2004, 2006). Implicit memory has been identified as a key functional region in flow states as it reduces verbal-analytical involvement in motor control by encouraging limited dependence on working memory (Masters, 1992; Maxwell et al., 2001; Liao and Masters, 2002) enabling performance with higher neural efficiency than explicit motor tasks relying on working memory (Zhu et al., 2011). Whereas, the automaticity reached in implicit memory is fast, effortless and free from distraction (Shiffrin and Schneider, 1977).

Specifically, the left dorsolateral prefrontal cortex (DLPFC) has been shown to modulate working memory (Barbey et al., 2013). Sharing Brodmann's area 8 (BA8) and close proximity to the frontal left is the medial prefrontal cortex (MPFC) which has been associated with self-monitoring and reflective processing employed during explicit processes which limit the efficiency of the system (Shiffrin and Schneider, 1977; Gusnard et al., 2001; Northoff et al., 2006; Yarrow et al., 2009). More recently, Ulrich et al. (2014) identified certain neural underpinnings that help explain part of the flow paradigm, in particular, a decrease in frontal activity around the MPFC.

Furthermore, the flow system is proposed to be a reflexive system guided by the preceding input (Dietrich, 2003). Therefore, it is believed that a basic level of skill acquisition is needed to have a flow experience, as the implicit system requires a series of learnt specialized and independent response patterns to output (Csikszentmihalyi and Csikszentmihalyi, 1988). These automated stimulus response procedures are believed to require many hours of highly dedicated practice. Learning of automated responses takes time because of the limited ability of the explicit working memory to transfer specialized and reflexive response patterns to the implicit system due to capacity restrictions (Mishkin et al., 1984; Dietrich, 2004). Experts are expected to have more automaticity available as the implicit system requires a series of specialized and independent response patterns to output, free from buffering other properties of the information in a higher order representation (Masters, 1992; Ohlsson, 2012). Flow is considered to increase in intensity on the continuum of experiential quality of the activity as the participant learns to utilize more of their dedicated facilities required for the task (Csikszentmihalyi and Csikszentmihalyi, 1988).

It has been shown that the brain makes use of an internal model which provides a sensorimotor representation of oneself with the world around (Jordan, 1996). Forward and inverse models can be utilized to explain the role of implicit processing by identifying the role of the network connecting the cerebellum, parietal and frontal regions to explain this control of high level processes such as decision making (Ito, 2008). These models consider that the prefrontal regions construct the mental model, but this mental model, used to explain and anticipate reality, exists in the parietal regions (Penfield and Perot, 1963), enabling the prefrontal region to be bypassed (Atherton et al., 2003; Chen et al., 2003). In one of the few neuroimaging studies on flow, an increase in activation was shown in the parietal regions as well as a decrease in prefrontal activity during a math task (Ulrich et al., 2014). Additionally, it has been shown that implicit bottom-up visual attention receives greater control from the parietal regions whereas top down control of more explicit processes are related to the frontal regions (Li et al., 2010). Furthermore, a long-range circuit has been found between these two regions that appears anatomically connected to guide choices toward movement goals (Sasaki et al., 1976; Pesaran et al., 2008).

To further test flow states and how it emerges, and possibly induced, is essential to better understand the flow state in practice. Transcranial direct current stimulation (tDCS) is a noninvasive brain stimulation technique that alters cortical excitability and activity in a polarity-dependent way. Anodal stimulation increases excitability (Liebetanz et al., 2002), whereas cathodal decreases it (Nitsche and Paulus, 2001). Stimulation for a few minutes has been shown to induce plastic alterations of cortical excitability and more specifically has shown to influence cognitive functions such as working memory by stimulating the left DLPFC (Fregni et al., 2005; Chrysikou et al., 2013; Zhu et al., 2015). Cathodal DLPFC tDCS has been shown to improve implicit learning outcomes for high-level motor tasks such as golf putting (Zhu et al., 2015) and cognitive flexibility (Chrysikou et al., 2013). Furthermore, it has been shown that tDCS has helped improve learning outcomes for implicit motor tasks, in which right parietal anodal stimulation resulted in greater neural efficiency through an improved task learning performance (Clark et al., 2012), as well as mental activities such as numerical competence (Cohen et al., 2010), network connectivity (Hunter et al., 2015) object detection during visual search (Bolognini et al., 2010; Clark et al., 2012; Tseng et al., 2012), spatial attention (Roy et al., 2015), and non-verbal material (Manuel and Schnider, 2016). Additionally, tDCS influence on parietal regions has shown a balance between the working memory capacity (skill) and the working memory task (Jones and Berryhill, 2012). More recently, Ulrich et al. (2018) used anodal tDCS over the forehead Fpz to stimulate the medial prefrontal cortex (MPFC) and found higher flow experiences for people experiencing low flow. Therefore, tDCS learning enhancement could increase the level of visual attention skill in order that the participant could reach the skill-challenge balance (Clark et al., 2012) and limit the role of the prefrontal monitoring in order to allow for greater movement into flow states (Zhu et al., 2015).

While flow states require a certain level of previous skill to be automatized into their implicit memory, tDCS has been shown to result in ceiling effects for experts compared to novice performers (Bullard et al., 2011; Tseng et al., 2012; Furuya et al., 2014; Rosen et al., 2016). Therefore, two groups of trained and untrained video gamers were selected for the study to explore the contrasting effects of the required skill acquisition and expertise to move into flow states with tDCS ceiling effects of expertise. The Tetris game paradigm has proved easy to quantify performance and level of difficulty in both flow (Keller and Bless, 2008; Keller et al., 2011; Harmat et al., 2015) and tDCS studies (Spiegel, 2013). First person perspective video games have also shown to operationalize a good balance of skill and challenge with immersive experiences for both flow (Kivikangas, 2006; Nacke L. and Lindley C., 2008; Nacke L. and Lindley C. A., 2008; Nacke and Lindley, 2010; Nacke et al., 2010; Klasen et al., 2011) and tDCS studies (Bullard et al., 2011; Clark et al., 2012; Coffman et al., 2012; Falcone and Parasuraman, 2012). Therefore, both experimental paradigms were used to determine the mediating role tDCS will have in supporting the induction of flow states.

The focus of this study was to observe the inductive role of tDCS on flow states using two different paradigms. It was hypothesized that right parietal anodal tDCS and cathodal tDCS of the left prefrontal area would result in a shift in the subjective experience toward higher intensity experiences of flow states for both trained and untrained users of video games.

## Materials and Methods

### Participants

Two experiments were ethically approved (by University Committee) to study the effects of tDCS on flow states during video game play. All participants were recruited by word of mouth or from advertisements in game forums. Experiment 1 inclusion requirement was trained gamers played 1st person shooter videogames (FPS) on average several times a week. Eleven right-handed males (*M* = 29 years, *SD* = 7.15) played a FPS across two sessions within a week using randomized active and sham tDCS conditions.

Experiment 2 inclusion requirement was untrained gamers who on average played videogames once a month or less. Twenty-three participants were originally tested but two were corrupted due to their being initial pilot tests, therefore only 21 right handed participants were tested; 11 females (*M* = 30.18 years, *SD* = 6.14) and 10 males (*M* = 31.8 years, *SD* = 3.61), played TETRIS® (Tetris Holding). Tetris was used for the untrained group as it is an easy game to learn and all participants were familiar with how to play it. Participants were randomly assigned between active and sham conditions.

### Inter-game Flow Questionnaire

At the end of each trial, participants were asked to retroactively assess their experience from their recent game trial and respond to a Flow State Scale (Jackson and Marsh, 1996) with two additional core questions of the flow state: “Everything Clicked” and “I was ‘in the zone’.”

### Game Play

In Experiment 1, participants were given the choice to play two different FPS games: “Counter Strike: Global Offensive” (Valve) or “Battlefield 4” (EA). Both games had the same settings of competing against live online players, most kills wins and played only in a single map environment. Due to different map, weapon and control settings, two games were used to allow players to participate in the FPS game they felt most proficient in to give them the best chance to enter into flow.

In Experiments 2, three versions of TETRIS were used: slow (bored), adaptive (flow), and fast (anxious). The slow round was set to a speed of 2 and the drop button was disabled, forcing the person to sit around and wait for the piece to reach the bottom of the screen. The anxious round started at speed level 8 and would go up once a person made 5 lines. The adaptive condition started at 4 and went up in score if the player made 5 lines in 20 moves, but it would slow a level down if they had not met this criterion.

### Stimulation

tDCS stimulation was applied using an NeuroConn DC-Stimulator (NeuroConn GmbH) machine with a montage of left prefrontal cathode and right parietal anode. tDCS was administered via two 5 × 5 cm electrodes covered with saline-soaked sponges. The stimulation site was determined by means of the 10/20 system, in which the cathode and anode were positioned over the F3 area and P6 area, respectively. Whilst tDCS excitability changes have been shown to last up to 60 min (Nitsche and Paulus, 2001), results have shown performance effects dwindle after 30 min of stimulation (Iyer et al., 2005). Therefore, stimulation condition was set for 20 min (including 10 s ramp-up and 10 s ramp-down time) at 2 mA while sham condition also lasted 20 min but was set for 30 s of stimulation at 1 mA. Participants are shown typically unable to determine whether receiving real or sham stimulation (Gandiga et al., 2006).

### Procedure

In Experiment 1, participants were told they were receiving tDCS stimulation over two separate sessions. In the first session, participants chose their FPS game and entered an online game room with 16 or more online players. The games' objective is to stop the other team therefore game scores were based on number of kills. Participants played a warm up round of free play without testing for about 20 min while the experiment set-up occurred. Participants would then be informed that testing would begin. A trial would last until the participant lasted longer than 3 min and completed two kills in a row without dying. They then would be notified the trial had finished with a flashing light controlled by the researcher to fill out the Inter-Game Flow Questionnaire. The participant would press a button to acknowledge the light flash before answering the questionnaire.

The participant was randomly assigned a stimulation or sham condition which lasted 20 min of either 2 mA for the active stimulation condition or 30 s of 1 mA over the 20 min period for sham condition. Participants would continue to play during that time without testing. Participants would then begin another testing session after stimulation following the previous testing procedure. Experiment 1 participants would return a week later and participate again with the same experimental protocol but receiving the opposite stimulation condition.

In Experiment 2, participants played a 15 min warm up of the balanced condition prior to testing. Then the participants would be informed about a change in the gaming condition and they would complete two trials of the slow, fast, and then adaptive TETRIS games for ~3 min. The researcher would then request they complete the Inter-Game Flow Questionnaire after each trial. The participant was randomly assigned a stimulation or sham condition which lasted 20 min of either 2 mA for the active stimulation condition or 30 s of 1 mA over the 20 min period for sham condition. Participants would continue to play the adaptive condition during that time, and complete subsequent Inter-Game Flow Questionnaires. Participants would then begin another testing session after stimulation but only complete the adaptive and fast conditions.

### Statistical Analysis

The research explored different hypotheses around performance ceilings as well as flow induction for the different training level of the groups to reduce learning effects and therefore enlisted different group design in the analysis.

#### Experiment 1

A repeated measures analysis of variance (ANOVA) was used to assess the significant main effect of the dependent variable, perceived state of flow score, during the first person video game before and after the two trials (tDCS and sham).

#### Experiment 2

A mixed ANOVA was used to determine a significant main effect of the dependent variable, perceived state of flow score, during the events associated with each of the trials and games; e.g., this was compared to lines completed in TETRIS during different conditions. Similarly, a mixed ANOVA was used to determine a significant main interaction effect for tDCS stimulation with each of the trials and games.

## Results

No participant reported experiencing adverse effects during or after tDCS. A slight itching sensation during approximately the first 30 s of stimulation was reported. The sham condition reported the same initial itching sensation, and when explicitly asked, believed to have undergone real stimulation.

**Figure 1:**
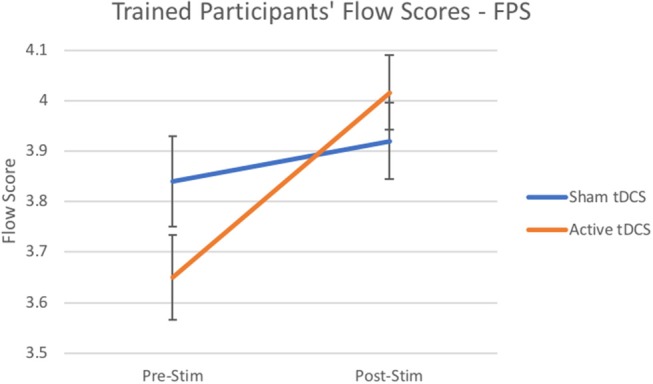
Flow scores from trained participants after Active Stimulation and Sham Stimulation. Bars—Standard Error.

An overall positive effect was observed for all participants from both experiments, in which participants from both experiments resulted in a significantly higher experience of flow states after tDCS compared to sham or control conditions. Experiment 1 hypothesized specifically that tDCS would modulate the experience of flow states for trained players of first-person shooter videogames. A repeated measures ANOVA determined a significant main effect of [*F*_(1, 54)_ = 5.82, *p* < 0.02, η_*p*_^2^ = 0.10; see Figure 1]. As hypothesized, simple main effects revealed that participants rated higher experiences of flow states after tDCS stimulation on average by (*M* = 0.37, *p* < 0.001, η_*p*_^2^ = 0.24) compared to sham which increased non-significantly on average by *M* = 0.08.

**Figure 2:**
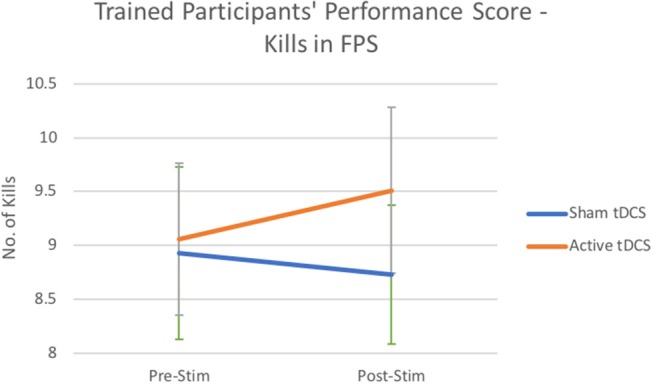
Number of kills performance scores from trained participants after Active Stimulation and Sham Stimulation. Bars—Standard Error.

Additionally, there were non-significant effects for main effects of kill performance [*F*_(1, 54)_ = 0.214, *p* = 0.645; see Figure 2], with greater performance improvements after tDCS on average by *M* = 0.45 compared to sham which reduced on average by *M* = −0.2.

**Figure 3:**
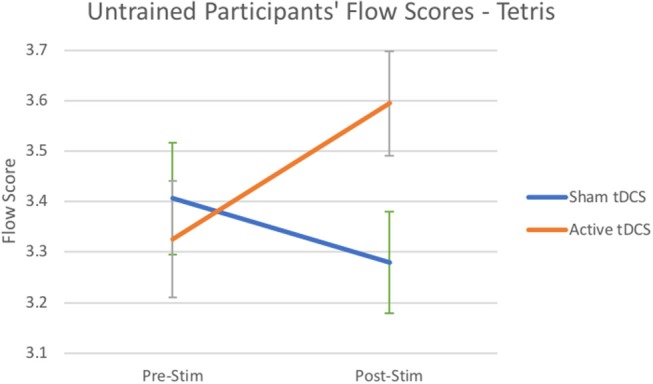
Flow scores from untrained participants playing TETRIS after Active Stimulation and Sham Stimulation. Bars—Standard Error.

Experiment 2 also resulted in the expected modulation pattern of flow states for untrained players of the puzzle game TETRIS. A mixed ANOVA was used to determine a significant main interaction effect for tDCS stimulation [*F*_(1, 48)_ = 7.24, *p* < 0.01, η_*p*_^2^ = 0.13; see Figure 3]. As hypothesized, planned simple main effects revealed participants in the flow condition rated higher experiences of flow states after tDCS stimulation on average by *M* = 0.27 (*p* < 0.02, η_*p*_^2^ = 0.22) compared to sham which reduced non-significantly by *M* = −0.13. While there was no main effect for the interaction of tDCS over time for the anxious condition, a significant effect showed higher flow states after tDCS stimulation by M = 0.27 (*p* < 0.05, η_*p*_^2^ = 0.2) compared to a non-significant effect for sham that increased flow scores on average by *M* = 0.17. Note that tDCS was not tested in the boredom condition.

**Figure 4:**
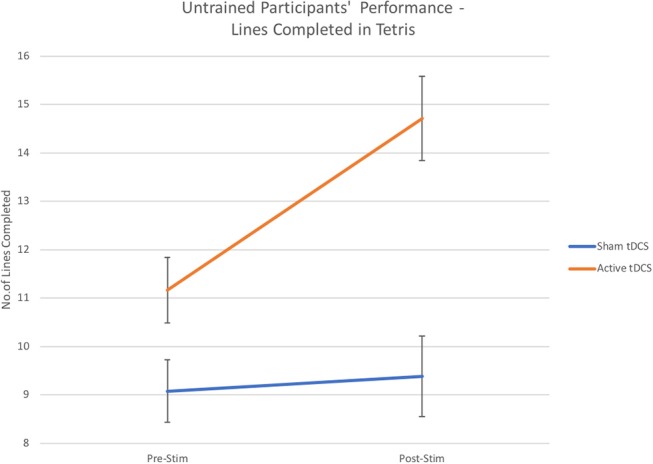
Number of completed lines—performance scores from untrained participants after Active Stimulation and Sham Stimulation. Bars—Standard Error.

Additionally, as expected there was a significant main interaction effect for performance in TETRIS based on number of completed lines [*F*_(1, 48)_ = 7.41, *p* < 0.01, η_*p*_^2^ = 0.13; see Figure 4], with greater line completion performance after tDCS on average by *M* = 3.54 (*p* < 0.001, η_*p*_^2^ = 0.4), compared to a non-significant effect for sham that increased line completion by *M* = 0.31.

## Discussion

As hypothesized, the results of this study indicate that tDCS can modulate an induction into flow states for video game players using a montage of prefrontal left cathode and right parietal anode. Additionally, as expected the trained FPS players performance was not improved by tDCS while the untrained TETRIS players improved due to tDCS stimulation compared to sham. While the results across both trained and untrained players of video games presented higher flow states after tDCS, the authors did find this interesting because it was unknown whether the performance ceiling effect might also effect the experienced intensity of flow states. While tDCS ceilings effects were present in the performance results of this study, which has been shown previously to apply to expert compared to novice performers (Bullard et al., 2011), studies have typically observed this from the perspective of motor skill tasks and not for psychological states. Perhaps psychological states may not be limited in the realm of performance by tDCS, i.e., tDCS studies have been shown to improve mood (Nitsche et al., 2009) and maybe further worth exploring the difference in limits tDCS modulation has for psychological states compared to motor skills. Another reason for the lack of ceiling effect may be that the high frequency of game play in the trained vs. the untrained group was not high enough to denote expertise and thus diminish the modulating effects of tDCS on flow states.

Whilst, to the authors knowledge, there has only been one prior research paper published on tDCS for flow states, which used a different montage of anodal stimulation over Fpz (Ulrich et al., 2018), the findings in this study could therefore be considered foreshadowed by previous papers documenting effects of tDCS in learning and working memory. The current findings align with previous research indicating that cathodal left prefrontal tDCS stimulation, as shown by Zhu et al. (2015), results in the reliance of improved implicit motor learning which could be considered to increase the modulation of the intensity of the flow experience as more resources are freed up for experiential processing (Dietrich, 2003). Inhibiting DLPFC has been shown to increase motor learning by disrupting the explicit motor system (Galea et al., 2010), as well as a dynamic balance with resources between explicit and implicit systems (Eichenbaum and Cohen, 2004; Kantak et al., 2012). The current study aimed to take advantage of this disruption of explicit executive functions to enhance the role of implicit processing and hence enable easier movement into elevated intensity of flow states. Furthermore, Zhu et al. (2015) reported a reduction in verbal working memory after the application of left prefrontal cathodal tDCS which Dietrich (2003) considers a requirement of his hypofrontality hypothesis to describe flow due to the reduction of high level buffering and maintenance.

Furthermore, the current findings also align with previous right anodal parietal research indicated in Clark et al. (2012) which resulted in positive learning effects in visual attention, thereby possibly reducing the amount of resources required to dedicate to the task to facilitate flow through implicit systems. Furthermore, the fronto-parietal attention network has been shown as a brain network relevant to attention activation during target detection tasks (Posner and Petersen, 1990). A review by Andersen and Cui (2009) indicated the role that the posterior parietal cortices (PPC) plays in the frontal parietal network through sensorimotor transformations including planning, decision making, forward model estimation and attentional faculties. Additionally, the tDCS has been shown to influence parietal regions based on a balance between the working memory capacity (skill) and the working memory task (Jones and Berryhill, 2012) which appears quite similar to the principle antecedents of flow states (Csikszentmihalyi and Csikszentmihalyi, 1988). In this study, we suspect that as attentional resources continue to increase during visual search elements of a task, such as video games, it may lead to a greater probability of noticing target objects, enhanced encoding of the location of the target object within the image and, therefore, greater accuracy and less buffering. This reduction in processing requirement could possibly open up the processing capacity to increase the perception of skill and thereby result in higher flow states ratings.

Dietrich (2004) originally considers flow states a reflexive system however from these results a new understanding maybe beginning to unfold as flow states may better be considered a predictive system that has developed and implemented through “forward and inverse models” which are considered neurological attempts at predicting the outcome of each action (Kawato, 1999). Ito (2008) describes the forward model through the prefrontal, temporal-parietal, and cerebellar network, in which the prefrontal area as the “controller” creates and transmits command signals that modify activities encoded while the temporo-parietal areas are considered the “mental model” which converts a command into an output action. Parietal anodal stimulation appeared to increase within network connectivity between key elements of the forward and inverse models including the inferior and superior parietal along with the cerebellar intrinsic networks, key for enhanced learning outcomes (Hunter et al., 2015).

This forward model could help explain the modulatory impact of the tDCS in inducing flow states as the system becomes less reliant on the moderating effects of the prefrontal controller whilst encouraging the ability to output commands fed in from the cerebellar network. This freedom from higher order interference enables the action output of the temporal-parietal regions the ability to more easily implement the memory model. This smoother activation free from frontal modulation may have resulted in the experience of less thinking and concern with the surroundings while the parietal excitation may have felt like an easier implementation of the memory models.

Additionally, the inverse model affords the prefrontal area to be bypassed and instead processing relies more heavily on the anterior cingulate cortex (ACC). The ACC has also been shown to be involved in flow states such as an EEG game study testing the difference between boredom, frustration and flow states (Nuñez Castellar et al., 2016). The ACC was determined as an actor in engaging the fronto-parietal network as well as monitoring conflicts in the focus of attention (Walsh et al., 2011). However, more recently Ulrich et al. (2014) found in a similar three level (boredom, flow, overload) arithmetic fMRI study of flow that the ACC reduced in activity. Nonetheless, while more study is needed to ascertain its role in attentional focus and flow states, the pattern of decreased prefrontal activity and increased parietal activity reported in Ulrich et al. (2014), found flow state results that mirrored the fronto-parietal network tDCS montage used in this study. It would be interesting to replicate this current study with a mirrored montage as the forward model appears to be supported by bilateral activation of the fronto-parietal network.

### Limitations

Whilst the results are indicative of a positive intervention of tDCS toward flow states, it would also be advantageous to consider the vast range of tDCS impacts. TDCS's effects have been shown as distributed rather than local (Keeser et al., 2011) and thus could impact unintended areas such that placing the prefrontal cathodal could influence multiple areas such as the DLPFC and the MPFC. Therefore, it may be worth considering using High Definition-tDCS in order to more accurately target locations associated with flow states in order to understand which areas specifically are responsible.

Furthermore, it is difficult to assess the full comparative impact of tDCS on flow and the ceiling effects between the trained and untrained players because the experimental design used a different methodology of a repeated experiment, alternating sham and tDCS for trained players while for untrained players they were only exposed one time to the experiment with a random allocation of tDCS or sham. This testing methodology in addition to testing between two different gaming paradigms are contributing factors to confounding the results. Therefore, for future testing it would be worth testing the role of tDCS ceiling effects on flow scores between trained and untrained players using the same experimental and gaming paradigm.

Additionally, it would be interesting to test different tDCS montages for modulating flow states. Flow states had been found in neuroimaging studies with both left and right parietal activation (Ulrich et al., 2014). Additionally, forward model neuroimaging studies have shown bilateral activation of parietal regions (Heinzel et al., 2017; Sokolowski et al., 2017).

## Conclusions

In the present study, we explored the subjective experiences of flow states for video gamers at different level of training after a tDCS intervention with a montage of a left prefrontal cathode and right parietal anode. Results revealed a subjective change toward higher intensity of flow experiences and an expected ceiling on task performance for trained and an improvement in task performance for untrained participants. With more research, tDCS could prove to be an effective tool to uncover more of the functional pathways involved in flow states and promote more positive subjective experiences for complex tasks including greater levels of immersion and enjoyment. By improving performance and states, tDCS could assist people to become more diligent, motivated and effective in tasks for occupational and rehabilitative efforts.

## Data Availability

The datasets generated for this study are available on request to the corresponding author.

## Ethics Statement

This study was carried out in accordance with the recommendations of Swinburne University Human Experimentation Committee (SUHREC) with written informed consent from all subjects. All subjects gave written informed consent in accordance with the Declaration of Helsinki. The protocol was approved by the SUHREC.

## Author Contributions

JC and JG: conceptualization and experimentation. JG: manuscript. JC: review and supervision.

### Conflict of Interest Statement

The authors declare that the research was conducted in the absence of any commercial or financial relationships that could be construed as a potential conflict of interest.

## References

[B1] Andersen R. A. Cui H. (2009). Intention, action planning, and decision making in parietal-frontal circuits. Neuron 63, 568–583. 10.1016/j.neuron.2009.08.028 19755101

[B2] Atherton M. Zhuang J. Bart W. M. Hu X. He S. (2003). A functional MRI study of high-level cognition. I. The game of chess. Brain research. Cogn. Brain Res. 16, 26–31. 10.1016/S0926-6410(02)00207-0 12589885

[B3] Barbey A. K. Koenigs M. Grafman J. (2013). Dorsolateral prefrontal contributions to human working memory. Cortex 49, 1195–1205. 10.1016/j.cortex.2012.05.022 22789779 PMC3495093

[B4] Bolognini N. Fregni F. Casati C. Olgiati E. Vallar G. (2010). Brain polarization of parietal cortex augments training-induced improvement of visual exploratory and attentional skills. Brain Res. 1349, 76–89. 10.1016/j.brainres.2010.06.053 20599813

[B5] Bullard L. M. Browning E. S. Clark V. P. Coffman B. A. Garcia C. M. Jung R. E.. (2011). Transcranial direct current stimulation's effect on novice versus experienced learning. Exp. Brain Res. 213, 9–14. 10.1007/s00221-011-2764-2 21706300

[B6] Chen X. Zhang D. Zhang X. Li Z. Meng X. He S.. (2003). A functional MRI study of high-level cognition: II. The game of GO. Brain Res. Cogn. Brain Res. 16, 32–37. 10.1016/S0926-6410(02)00206-9 12589886

[B7] Chrysikou E. G. Hamilton R. H. Coslett H. B. Datta A. Bikson M. Thompson-Schill S. L. (2013). Noninvasive transcranial direct current stimulation over the left prefrontal cortex facilitates cognitive flexibility in tool use. Cogn. Neurosci. 4, 81–89. 10.1080/17588928.2013.768221 23894253 PMC3719984

[B8] Clark V. P. Coffman B. A. Mayer A. R. Weisend M. P. Lane T. D. R. Calhoun V. D.. (2012). TDCS guided using fMRI significantly accelerates learning to identify concealed objects. Neuroimage 59, 117–128. 10.1016/j.neuroimage.2010.11.036 21094258 PMC3387543

[B9] Coffman B. A. Trumbo M. Flores R. Garcia C. M. Van Der Merwe A. Wassermann E. M.. (2012). Impact of tDCS on performance and learning of target detection: interaction with stimulus characteristics and experimental design. Neuropsychologia 50, 1594–1602. 10.1016/j.neuropsychologia.2012.03.012 22450198

[B10] Cohen K. R. Soskic S. Iuculano T. Kanai R. Walsh V. (2010). Modulating neuronal activity produces specific and long-lasting changes in numerical competence. Curr. Biol. 20, 2016–2020. 10.1016/j.cub.2010.10.007 21055945 PMC2990865

[B11] Csikszentmihalyi I. Csikszentmihalyi M. (1988). Optimal Experience: Psychological Studies of Flow in Consciousness. New York, NY: Cambridge University Press 10.1017/CBO9780511621956

[B12] Csikszentmihalyi M. (1990). Flow: The Psychology of Optimal Experience. New York, NY: Harper & Row.

[B13] Csikszentmihalyi M. (1997). Finding Flow: The Psychology of Engagement With Everyday Life. New York, NY: Basic Books.

[B14] Csikszentmihalyi M. Nakamura J. (1999). Emerging goals and the self-regulation of behavior. Adv. Soc. Cogn. 12, 107–118.

[B15] Dietrich A. (2003). Functional neuroanatomy of altered states of consciousness: the transient hypofrontality hypothesis. Conscious. Cogn. 12, 231–256. 10.1016/S1053-8100(02)00046-6 12763007

[B16] Dietrich A. (2004). Neurocognitive mechanisms underlying the experience of flow. Conscious. Cogn. 13, 746–761. 10.1016/j.concog.2004.07.002 15522630

[B17] Dietrich A. (2006). Transient hypofrontality as a mechanism for the psychological effects of exercise. Psychiatry Res. 145, 79–83. 10.1016/j.psychres.2005.07.033 17081621

[B18] Eichenbaum H. Cohen N. J. (2004). From Conditioning to Conscious Recollection: Memory Systems of the Brain. Oxford: Oxford University Press 10.1093/acprof:oso/9780195178043.001.0001

[B19] Falcone B. Parasuraman R. (2012). Comparative effects of first-person shooter video game experience and brain stimulation on threat detection learning, in Paper Presented at the Proceedings of the Human Factors and Ergonomics Society Annual Meeting (London: SAGE Publications). 10.1177/1071181312561013

[B20] Fregni F. Boggio P. S. Nitsche M. Bermpohl F. Antal A. Feredoes E.. (2005). Anodal transcranial direct current stimulation of prefrontal cortex enhances working memory. Exp. Brain Res. 166, 23–30. 10.1007/s00221-005-2334-6 15999258

[B21] Furuya S. Klaus M. Nitsche M. A. Paulus W. Altenmüller E. (2014). Ceiling effects prevent further improvement of transcranial stimulation in skilled musicians. J. Neurosci. 34, 13834–13839. 10.1523/JNEUROSCI.1170-14.2014 25297109 PMC6608385

[B22] Galea J. M. Albert N. B. Ditye T. Miall R. C. (2010). Disruption of the dorsolateral prefrontal cortex facilitates the consolidation of procedural skills. J. Cogn. Neurosci. 22, 1158–1164. 10.1162/jocn.2009.21259 19413472 PMC6010144

[B23] Gandiga P. C. Hummel F. C. Cohen L. G. (2006). Transcranial DC stimulation (tDCS): a tool for double-blind sham-controlled clinical studies in brain stimulation. Clin. Neurophysiol. 117, 845–850. 10.1016/j.clinph.2005.12.003 16427357

[B24] Gusnard D. A. Akbudak E. Shulman G. L. Raichle M. E. (2001). Medial prefrontal cortex and self-referential mental activity: relation to a default mode of brain function. Proc. Natl. Acad. Sci. U.S.A. 98, 4259–4264. 10.1073/pnas.071043098 11259662 PMC31213

[B25] Harmat L. de Manzano Ö. Theorell T. Högman L. Fischer H. Ullén F. (2015). Physiological correlates of the flow experience during computer game playing. Int. J. Psychophysiol. 97, 1–7. 10.1016/j.ijpsycho.2015.05.001 25956190

[B26] Heinzel S. Rimpel J. Stelzel C. Rapp M. A. (2017). Transfer effects to a multimodal dual-task after working memory training and associated neural correlates in older adults–a pilot study. Front. Hum. Neurosci. 11:85. 10.3389/fnhum.2017.00085 28286477 PMC5323430

[B27] Hunter M. A. Coffman B. A. Gasparovic C. Calhoun V. D. Trumbo M. C. Clark V. P. (2015). Baseline effects of transcranial direct current stimulation on glutamatergic neurotransmission and large-scale network connectivity. Brain Res. 1594, 92–107. 10.1016/j.brainres.2014.09.066 25312829 PMC4358793

[B28] Ito M. (2008). Control of mental activities by internal models in the cerebellum. Nat. Rev. Neurosci. 9, 304–313. 10.1038/nrn2332 18319727

[B29] Iyer M. Mattu U. Grafman J. Lomarev M. Sato S. Wassermann E. (2005). Safety and cognitive effect of frontal DC brain polarization in healthy individuals. Neurology 64, 872–875. 10.1212/01.WNL.0000152986.07469.E9 15753425

[B30] Jackson S. A. Marsh H. W. (1996). Development and validation of a scale to measure optimal experience: the flow state scale. J. Sport Exerc. Psychol. 18, 17–35. 10.1123/jsep.18.1.17

[B31] Jones K. T. Berryhill M. E. (2012). Parietal contributions to visual working memory depend on task difficulty. Front. Psychiatry 3:81. 10.3389/fpsyt.2012.00081 22973241 PMC3437464

[B32] Jordan M. I. (1996). Computational aspects of motor control and motor learning, in Handbook of Perception and Action, Vol. 2, eds Heuer H. Keele S. W. (Amsterdam: Elsevier), 71–120. 10.1016/S1874-5822(06)80005-8

[B33] Kantak S. S. Mummidisetty C. K. Stinear J. W. (2012). Primary motor and premotor cortex in implicit sequence learning – evidence for competition between implicit and explicit human motor memory systems. Eur. J. Neurosci. 36, 2710–2715. 10.1111/j.1460-9568.2012.08175.x 22758604

[B34] Kawato M. (1999). Internal models for motor control and trajectory planning. Curr. Opin. Neurobiol. 9, 718–727. 10.1016/S0959-4388(99)00028-8 10607637

[B35] Keeser D. Meindl T. Bor J. Palm U. Pogarell O. Mulert C.. (2011). Prefrontal transcranial direct current stimulation changes connectivity of resting-state networks during fMRI. J. Neurosci. 31, 15284–15293. 10.1523/JNEUROSCI.0542-11.2011 22031874 PMC6703525

[B36] Keller J. Bless H. (2008). Flow and regulatory compatibility: an experimental approach to the flow model of intrinsic motivation. Pers. Soc. Psychol. Bull. 34, 196–209. 10.1177/0146167207310026 18212330

[B37] Keller J. Ringelhan S. Blomann F. (2011). Does skills–demands compatibility result in intrinsic motivation? Experimental test of a basic notion proposed in the theory of flow-experiences. J. Posit. Psychol. 6, 408–417. 10.1080/17439760.2011.604041

[B38] Kivikangas J. M. (2006). Psychophysiology of Flow Experience: An Explorative Study. Helsinki: University of Helsinki.

[B39] Klasen M. Weber R. Kircher T. T. Mathiak K. A. Mathiak K. (2011). Neural contributions to flow experience during video game playing. Soc. Cogn. Affect. Neurosci. 7, 485–495. 10.1093/scan/nsr021 21596764 PMC3324568

[B40] Li L. Gratton C. Yao D. Knight R. T. (2010). Role of frontal and parietal cortices in the control of bottom-up and top-down attention in humans. Brain Res. 1344, 173–184. 10.1016/j.brainres.2010.05.016 20470762 PMC2900444

[B41] Liao C.-M. Masters R. S. W. (2002). Self-focused attention and performance failure under psychological stress. J. Sport Exerc. Psychol. 24, 289–305. 10.1123/jsep.24.3.289 28682203

[B42] Liebetanz D. Nitsche M. A. Tergau F. Paulus W. (2002). Pharmacological approach to the mechanisms of transcranial DC-stimulation-induced after-effects of human motor cortex excitability. Brain 125, 2238–2247. 10.1093/brain/awf238 12244081

[B43] Manuel A. L. Schnider A. (2016). Effect of prefrontal and parietal tDCS on learning and recognition of verbal and non-verbal material. Clin. Neurophysiol. 127, 2592–2598. 10.1016/j.clinph.2016.04.015 27291878

[B44] Masters R. S. W. (1992). Knowledge, knerves and know-how: the role of explicit versus implicit knowledge in the breakdown of a complex motor skill under pressure. Br. J. Psychol. 83, 343–358. 10.1111/j.2044-8295.1992.tb02446.x

[B45] Maxwell J. P. Masters R. S. Kerr E. Weedon E. (2001). The implicit benefit of learning without errors. Q. J. Exp. Psychol. Sect. A 54, 1049–1068. 10.1080/713756014 11765732

[B46] Mishkin M. Malamut B. Bachevalier J. (1984). Memory and habit: two neural systems, in Neurobiology of Learning and Memory, eds Lynch G. McGaugh J. J. Weinberger N. M. (New York, NY: Guilford Press, 66–77.

[B47] Nacke L. Lindley C. (2008). Boredom, Immersion, Flow: a pilot study investigating player experience, in Paper Presented at the IADIS International Conference Gaming 2008: Design for Engaging Experience and Social Interaction (Amsterdam: IADIS Press).

[B48] Nacke L. Lindley C. A. (2008). Flow and immersion in first-person shooters: measuring the player's gameplay experience, in Paper presented at the Proceedings of the 2008 Conference on Future Play: Research, Play, Share (Ithaca, NY: Cornell University). 10.1145/1496984.1496998

[B49] Nacke L. E. Lindley C. A. (2010). Affective ludology, flow and immersion in a first-person shooter: measurement of player experience. arXiv [preprint]. arXiv:1004.0248.

[B50] Nacke L. E. Stellmach S. Lindley C. A. (2010). Electroencephalographic assessment of player experience: a pilot study in affective ludology. arXiv preprint arXiv:1004.0248. 10.1177/1046878110378140

[B51] Nakamura J. Csikszentmihalyi M. (2014). The concept of flow, in Flow and the Foundations of Positive Psychology (Dordrecht: Springer), 239–263. 10.1007/978-94-017-9088-8_16

[B52] Nitsche M. A. Boggio P. S. Fregni F. Pascual-Leone A. (2009). Treatment of depression with transcranial direct current stimulation (tDCS): a review. Exp. Neurol. 219, 14–19. 10.1016/j.expneurol.2009.03.038 19348793

[B53] Nitsche M. A. Paulus W. (2001). Sustained excitability elevations induced by transcranial DC motor cortex stimulation in humans. Neurology 57, 1899–1901. 10.1212/WNL.57.10.1899 11723286

[B54] Northoff G. Heinzel A. de Greck M. Bermpohl F. Dobrowolny H. Panksepp J. (2006). Self-referential processing in our brain—A meta-analysis of imaging studies on the self. Neuroimage 31, 440–457. 10.1016/j.neuroimage.2005.12.002 16466680

[B55] Nuñez Castellar E. P. Antons J.-N. Marinazzo D. van Looy J. (2016). Being in the zone: using behavioral and EEG recordings for the indirect assessment of flow. PeerJ Preprints 4:e24821 10.7287/peerj.preprints.2482v1

[B56] Ohlsson S. (2012). Cognitive skill acquisition, in Encyclopedia of the Sciences of Learning, ed Seel N. M. (Boston, MA: Springer US), 616–619. 10.1007/978-1-4419-1428-6_297

[B57] Penfield W. Perot P. (1963). The brains record of auditory and visual experience 1: a final summary and discussion. Brain 86, 595–696. 10.1093/brain/86.4.595 14090522

[B58] Pesaran B. Nelson M. J. Andersen R. A. (2008). Free choice activates a decision circuit between frontal and parietal cortex. Nature 453, 406–409. 10.1038/nature06849 18418380 PMC2728060

[B59] Posner M. I. Petersen S. E. (1990). The attention system of the human brain. Annu. Rev. Neurosci. 13, 25–42. 10.1146/annurev.ne.13.030190.000325 2183676

[B60] Rosen D. S. Erickson B. Kim Y. E. Mirman D. Hamilton R. H. Kounios J. (2016). Anodal tDCS to right dorsolateral prefrontal cortex facilitates performance for novice jazz improvisers but hinders experts. Front. Hum. Neurosci. 10:579. 10.3389/fnhum.2016.00579 27899889 PMC5110534

[B61] Roy L. B. Sparing R. Fink G. R. Hesse M. D. (2015). Modulation of attention functions by anodal tDCS on right PPC. Neuropsychologia 74, 96–107. 10.1016/j.neuropsychologia.2015.02.028 25721567

[B62] Sasaki K. Kawaguchi S. Oka H. Sakai M. Mizuno N. (1976). Electrophysiological studies on the cerebellocerebral projections in monkeys. Exp. Brain Res. 24, 495–507. 10.1007/BF00234966 1253863

[B63] Shiffrin R. M. Schneider W. (1977). Controlled and automatic human information processing: II. Perceptual learning, automatic attending and a general theory. Psychol. Rev. 84, 127–190. 10.1037/0033-295X.84.2.127

[B64] Sokolowski H. M. Fias W. Mousa A. Ansari D. (2017). Common and distinct brain regions in both parietal and frontal cortex support symbolic and nonsymbolic number processing in humans: a functional neuroimaging meta-analysis. Neuroimage 146, 376–394. 10.1016/j.neuroimage.2016.10.028 27769786

[B65] Spiegel D. (2013). Transcranial direct current stimulation of the healthy and amblyopic visual cortex: mechanisms and action (Doctoral theses). The University of Auckland, Auckland, New Zealand.

[B66] Tseng P. Hsu T.-Y. Chang C.-F. Tzeng O. J. Hung D. L. Muggleton N. G.. (2012). Unleashing potential: transcranial direct current stimulation over the right posterior parietal cortex improves change detection in low-performing individuals. J. Neurosci. 32, 10554–10561. 10.1523/JNEUROSCI.0362-12.2012 22855805 PMC6621415

[B67] Ulrich M. Keller J. Hoenig K. Waller C. Grön G. (2014). Neural correlates of experimentally induced flow experiences. Neuroimage 86, 194–202. 10.1016/j.neuroimage.2013.08.019 23959200

[B68] Ulrich M. Niemann J. Boland M. Kammer T. Niemann F. Grön G. (2018). The neural correlates of flow experience explored with transcranial direct current stimulation. Exp. Brain Res. 236, 3223–3237. 10.1007/s00221-018-5378-0 30209517

[B69] Walsh B. J. Buonocore M. H. Carter C. S. Mangun G. R. (2011). Integrating conflict detection and attentional control mechanisms. J. Cogn. Neurosci. 23, 2211–2221. 10.1162/jocn.2010.21595 21126158 PMC3142580

[B70] Yarrow K. Brown P. Krakauer J. W. (2009). Inside the brain of an elite athlete: the neural processes that support high achievement in sports. Nat. Rev. Neurosci. 10, 585–596. 10.1038/nrn2672 19571792

[B71] Zhu F. F. Poolton J. M. Wilson M. R. Hu Y. Maxwell J. P. Masters R. S. (2011). Implicit motor learning promotes neural efficiency during laparoscopy. Surg. Endosc. 25:2950. 10.1007/s00464-011-1647-8 21455805 PMC3160550

[B72] Zhu F. F. Yeung A. Y. Poolton J. M. Lee T. M. C. Leung G. K. K. Masters R. S. (2015). Cathodal transcranial direct current stimulation over left dorsolateral prefrontal cortex area promotes implicit motor learning in a golf putting task. Brain Stimul. 8, 784–786. 10.1016/j.brs.2015.02.005 25857398

